# Parentage-based tagging and parentage analyses of stocked sea trout in Vistula River commercial catches

**DOI:** 10.1007/s13353-023-00749-9

**Published:** 2023-02-06

**Authors:** Anna Wąs-Barcz, Rafał Bernaś

**Affiliations:** 1 National Marine Fisheries Research Institute, Department of Fisheries Resources, Kołłątaja 1, 81-332 Gdynia, Poland; 2 National Inland Fisheries Research Institute, Department of Migratory Fish, Rutki 49, 83-330 Żukowo, Poland

**Keywords:** Sea trout, Stocking, Vistula River, Hatchery broodstock, Parentage analysis, Spawning migration

## Abstract

The longest Baltic River, the Vistula, historically hosted numerous sea trout populations. However, dam construction in the twentieth century drastically reduced the spawning migration rate. Reduced natural reproduction has resulted in a population collapse and, consequentially, a substantial reduction in catches. In response, like other Baltic countries, Poland has initiated an intensive stocking program, mainly involving smolt. Initially, stocking was conducted primarily with offspring of sea trout caught during spawning migration. Currently, due to difficulties in obtaining fish, most stocking involves fish from breeding stocks. Therefore, determining the proportion of fish derived from stocking has become an important issue. Experiments based on traditional tagging did not provide sufficient material for analysis; hence, we decided to use genetic methods based on analysis of relatedness. In this study, we performed parentage-based tagging and an analysis of the origins of parent animals used for artificial spawning in 2013, and offspring returning to the Vistula in subsequent years. We based the analysis on three different algorithms and compared the results, showing that the presented methods were effective for estimating mass stocking success. The study also indicated that a certain level of natural reproduction in the Vistula continues to occur. The proportion of sea trout from spawning in 2013 in Vistula sea trout catches from 2017 to 2018 was approximately 30%.

**Supplementary Information:**

The online version contains supplementary material available at 10.1007/s13353-023-00749-9.

## Introduction

**Fig. 1:**
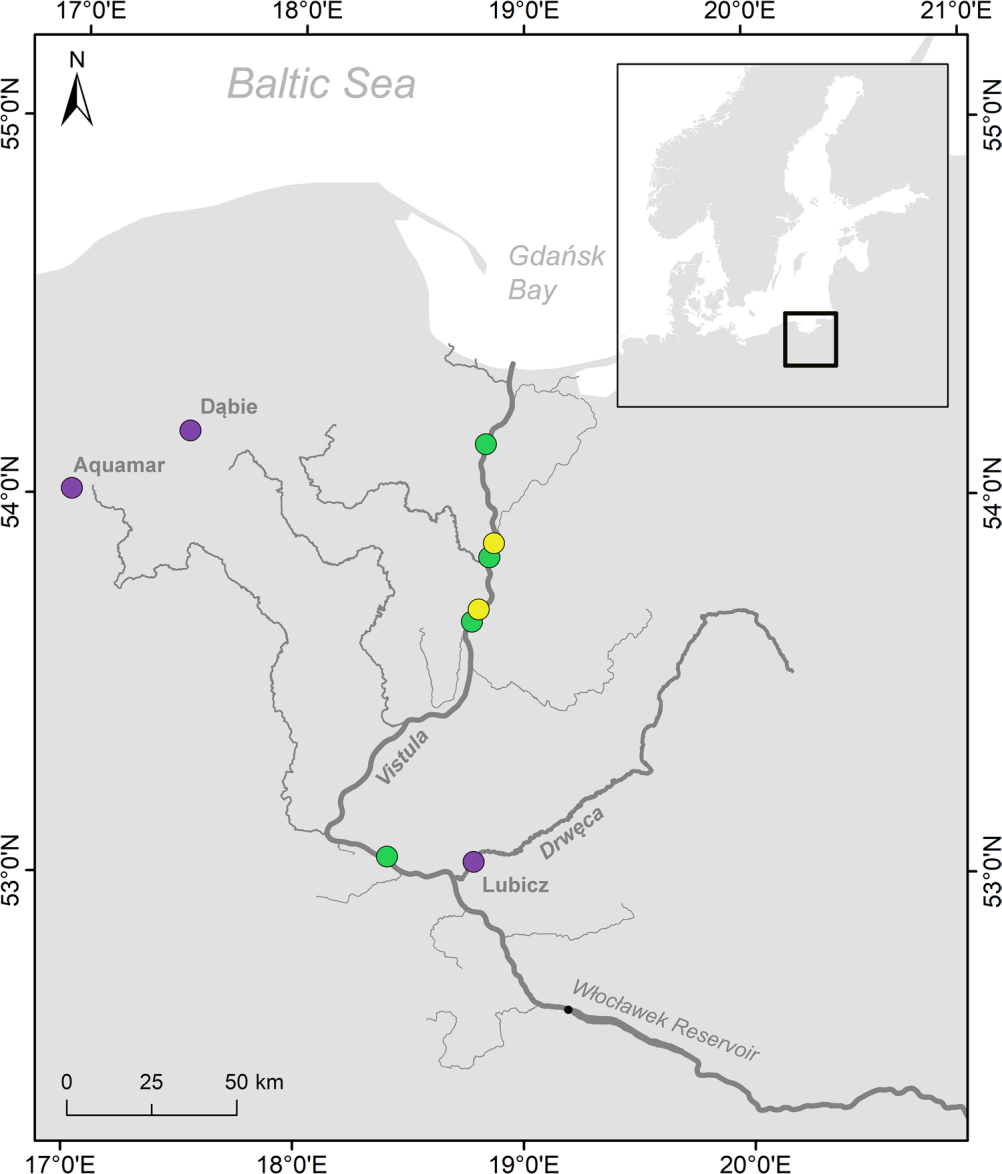
Sea trout sampling sites from the Vistula River commercial catches in 2017 (green dots) and 2018 (yellow dots). Violet dots represent localisation of spawners sampling sites from artificial spawning in 2013

Brown trout (*Salmo trutta* L.) is the most widely distributed freshwater fish native to the Palearctic region. Its natural range extends from northern Europe to North Africa and from Iceland to the headwaters of Aral Sea from west to east (Bernatchez 2001). This species is polymorphic and has several life strategies. The anadromous form is referred to as migratory sea trout, and it migrates from its natal stream to the sea to feed and grow before returning to its birthplace to spawn. By contrast, the resident form known as brown trout spends its entire life in freshwater and often spawns in the smaller tributaries of the stream it inhabits (Elliott 1994). Nowadays, there are ~ 400 migratory sea trout populations in the Baltic Sea (ICES 2021). Poland has ~ 25 sea trout rivers in which this species spawns naturally, and these are mainly in the Pomeranian region, but they are also located in the Vistula and Oder River drainage basins (HELCOM 2011). Historically, the largest population of sea trout in the Baltic catchment area was found in the Vistula River (Dębowski 2018). The Vistula River, the longest river flowing into the Baltic Sea, has the second highest run-off after the Neva River (BACC II Author Team 2015). In the past, the main spawning grounds were in the Carpathian area, ~ 1000 km from the Vistula river mouth in the Gdańsk Bay (Bernaś et al. 2020). Artificial stocking of the Vistula River began in the mid-nineteenth century, and most stocking used sea trout originating from spawners collected in the Dunajec River ~ 800 km from the Vistula mouth (Kołder, 1958). These fish stocks were irregular at least until the 1940s, and they grew over time. Progressive hydrotechnical development in the upper Vistula basin resulted in difficulties obtaining spawners, and extreme population collapse began in 1968 when Włocławek Dam was built in the middle course of the Vistula (Fig. 1). Spawners were stopped below the dam, and from that time, the best reproductive area in the mountain tributaries was lost for many years. Since construction of the dam in Włocławek was finished in 1969, collection of spawners in the upper segment of the river has been unsuccessful (Dębowski 2018).

Sea trout stocking has a long tradition in Baltic countries and has been associated with aquaculture development and increasing catches since the 1960s. The maximum total catches were in the years 1985-1995, with those years averaging ~ 1300 tons per year. By contrast, in the last 10 years, the average was only ~ 600 tonnes per year (ICES 2021), even though stocking levels have been increasing. In the years of the highest catches (1985-1995), slightly over two million smolts were released per year, and in the last 10 years, it was significantly above 3 million. This disproportion between the number of stocked and caught fish has many causes, including improved fishing efficiency, post-smolt mortality, food availability and predation (Mäntyniemi et al. 2012). But the effectiveness of stocking remains poorly understood, including the potentially damaging effects of stocked fish on wild populations (e.g. Araki et al. 2008; Naish et al. 2007). In order to explore this, methods of estimating the effectiveness of stocking have been developed. In the second half of the twentieth century, the most common marking method for Baltic salmonids was either the external Carlin tag or the FloyTag (Bartel et al. 2010; Drenner et al. 2012). This type of marking is still used, albeit on a very limited scale, due to successively diminishing tag returns, which currently do not permit assessment of effectiveness (ICES 2021). Additionally, fish size is a limitation of this type of tagging (Nielsen 1992). Marking methods for larvae or fry include immersion in fluorescent dyes (Secor et al. 1991; Jones et al. 2005), using transgenerational enriched stable isotopes that appear in bone tissue (Munro et al. 2009), or chemicals such as oxytetracycline (Krumme and Bingel 2016). In the last 20 years, adipose fin clipping (Petersson et al. 2014) has become the dominant form of tagging for sea trout and salmon in the Baltic catchment. The total number of fin-clipped sea trout released in 2020 in the Baltic Sea area was > 1.3 million. Fin-clipping of hatchery-reared smolts is mandatory in Sweden, Finland and Estonia (ICES 2021). However, this tagging method is currently not used in Poland due to ethical and veterinary concerns.

The development of molecular techniques using highly polymorphic loci (microsatellite DNA or SNPs) has allowed the development of molecular tools for estimating stocking efficiency. These methods are based on identification of individual genotypes and their variability (Estoup et al. 1998). The usefulness of genetic methods in analysing parentage has been confirmed for many species including Atlantic salmon, rainbow trout, Atlantic cod, carp and brown trout (Norris and Cunningham 2004; Fishback et al. 2002; Vandeputte et al. 2004; Herlin et al. 2007; Wąs-Barcz et al. 2017).

The main goal of this study was to estimate the proportion of artificial breeding sea trout from spawning season 2013 in Vistula River catches in 2017-2018 using parentage-based tagging and parentage analyses with three different algorithms. To our knowledge, this study represents one of the largest sibship reconstruction attempts based on empirical data conducted to date.

## Materials and methods

### Study area

The River Vistula is the biggest river in Poland, and 86% of the catchment area covers over half of the area of the country. The river is 1020-km long and the size of the catchment area is 199,813 km^2^ with average flow of 1054 m^3^/s. Nowadays, the Vistula is fully accessible to migrating fish up to the Włocławek hydroelectric power plant. The dam constructed in 1968 has a fish ladder, rebuilt in 2015. Most large and medium-sized tributaries of the Lower Vistula are accessible to anadromous species only on short stretches. The exception is the largest tributary, the Drwęca River, which, thanks to the fish pass in Lubicz, is available for a considerable section (Fig. 1). On the Drwęca River, ascending sea trout are caught every year, the offspring of which are used for stocking in the basin.

In the case of the Vistula, stocking has been based on fish from breeding stocks for several years. In the period analysed, broodstock was from Aquamar and Dąbie fish farms. Aquamar farm began delivering stocking material for release in the early 2000s. In last decade, these fish comprised between 12 and 63% (average 25%) of the material released into the Vistula River (Dębowski 2018). However, in the last 5 years, this share has grown to ~40%. Dąbie fish farm was created in 2006 based on sea trout collected close to the Vistula mouth. It was the source of stocking material from 2009, and in recent years, these fish comprised about 30-40% of fish released (Bernaś et al. 2020). The sizes of broodstocks were 1000-1200 active females from Aquamar farm and ~ 600 females from Dąbie farm. Males constituted ~ 30% of this number. Broodstock renewals were maintained at ~ 10% per year. These farms are the largest producers of Vistula sea trout stocking materials.

### Sampling

Sampling began in autumn 2013 with the collection of genetic material (fin clip) from all sea trout spawners caught on the Drwęca River in Lubicz, used for artificial spawning (Fig. 1, Table 1). In the same period, samples were taken from all sea trout spawners used for spawning in 2013 in breeding stocks at Aquamar and Dąbie fish farms.

**Table 1:** Sea trout sample collection from 2013 spawners and fish returning to the Vistula River in 2017-2018

Year	Place	River/strain	Source	Number
2013	Lubicz	Drwęca	River fisheries	217
2013	Aquamar	Vistula	Broodstock	1115
2013	Dąbie	Vistula	Broodstock	433
2017	Wisła	Vistula	River fisheries	74
2018	Wisła	Vistula	River fisheries	381

**Fig. 2:**
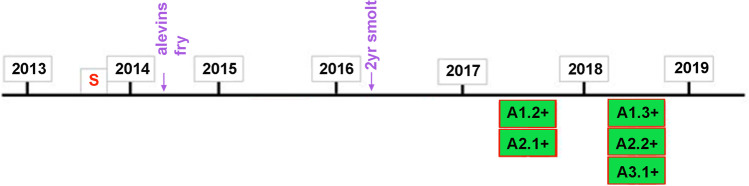
Experiment time line. Green rectangles refer to the age of sea trout sampled in 2017–2018 in the Vistula River that may have come from artificial spawning (S) in 2013. The first digits indicate the freshwater age, and the second digits represent the sea age. Vertical purple arrows indicate releases of spawning material produced in 2013

In total, parental material in 2013 was collected from 1765 individuals (Table 1). Offspring from sampled parents were released into the Vistula River and its tributaries in 2014 as alevin and fry (3,080,700 individuals) and as 2-year-old smolt in 2016 (619,100 individuals; Fig. 2). Subsequently, samples were collected from the sea trout which returned to Vistula in 2017 and 2018. Fish were caught using drifting gill nets by commercial fishers in the Vistula River and in trap located in the fishway near Lubicz in Drwęca River. Most of the returning fish were also measured to the nearest 0.5 cm total length (TL), weighed, and age was determined from collected scales (Table S1 and S2) as given in Bernaś et al. (2019). Sea age is understood as the growth period in marine waters as opposed to the growth period in freshwater (Sych 1967). Age determination, if possible, was used to verify the obtained parental detections. The experiment time line is shown in Fig. 2.

### Microsatellite genotyping

In total, fin clip samples (approximately 2–5 mm^2^) were collected from 2220 individual fish that were examined. Genomic DNA was extracted from fin tissue preserved in 96% ethanol using a Genomic Mini Kit (A&A Biotechnology, Gdynia, Poland) and diluted to a concentration of 30-100 ng μ^–1^. A set of 13 fluorescently labelled polymorphic microsatellite loci—*OneU9*, *Strutta58P*, *Ssosl438*, *Ssosl311*, *Str15INRA*, *Str543INRA*, *Str60INRA*, *Str73INRA*, *Ssosl417*, *Str85INRA*, *Ssa85*, *Bs131* and *Ssa407*—were amplified by single multiplex PCR using a Qiagen Multiplex PCR Kit (Qiagen, Düsseldorf, Germany). Each 10-μl multiplex PCR consisted of ~ 100 ng of template DNA, 1 × multiplex PCR master mix and 0.2-0.6 μM of each primer. Amplifications were carried out using a Professional Basic Gradient thermal cycler (Biometra, Göttingen, Germany) involving denaturation at 95 °C for 5 min followed by 38 cycles of denaturation at 94 °C for 30 s, annealing at 55 °C for 90 s and extension at 72 °C for 60 s. Reactions were terminated after 30 min, and the final extension was performed at 60 °C. PCR products were genotyped using single capillary electrophoresis on an ABI Prism 3130xl Genetic Analyzer (Applied Biosystems, Waltham, USA) along with GeneScan 600LIZ size standards (Applied Biosystems). DNA fragments were estimated using a Peak Scanner v1.0 (Applied Biosystems). Details regarding locus sources, concentrations and labelling of primers are provided in Wąs and Bernaś (2016), with the exception of *Ssa407* which was from Cairney et al. (2000); primer concentration was 0.4 pmol/μl, and fluorescent labelling was PET. The effectiveness of applied multiplex with 13 microsatellite loci in parentage analysis was previously tested using negative control and was found useful for Polish populations of sea trout (Wąs-Barcz et al. 2017).

### Data analysis

Verification of the relationship between fish returning to the Vistula in 2017-2018 and potential parents participating in artificial spawning in 2013 carried out with the use of three different algorithms. The first algorithm, the Family Assignment Program (FAP) (Taggart 2007), is based on the method of exclusion and calculated based on probability assuming all parental genotypes were known, which corresponded to our sampling regime. The program also offered useful tools that take into account the presence of mutations in offspring genotype and problematic loci generating errors when reading the genotype (misscoring phenomenon). The analyses were conducted assuming errors of 0 and 1 in genotype reading, as suggested by the authors of the program. This allowed for assigning offspring, in accordance with Mendelian law, as parent pairs with 100% matching genotypes, or it was possible to differentiate one locus within the complex parental genotype.

The second approach was based on an algorithm implemented in COLONY 2.0.6.6. (Jones and Wang 2010). We applied the full-likelihood (FL) method, medium run length and high precision. A genotyping error rate of 0.001 was employed as described previously (Palm et al. 2008). Due to the assumption that at least one of the parents is known, the analysis assigned parent pairs or one of the parents. The accepted probability threshold was *p* < 0.01.

The third algorithm based on the Bayesian approach was implemented using the SOLOMON R package (Christie et al. 2013). The values recommended for microsatellite markers were applied to 50,000,000 simulated datasets, assuming error-free genotype readings. Due to the occurring multigenerational connections and the potential high level of kinship, the acceptance threshold for the putative parent-offspring relationship was applied at the *p* < 0.01 level for certain relationships and *p* < 0.05 for probable ones. After calculation, the results obtained by the three methods were compared.

In order to describe each source for spawning in 2013 (broodstocks and spawners from Lubicz) in terms of family structure and abundance of genitors, parentage structure was assessed using Colony 2.0.6.6. (Jones and Wang 2010). We applied non-default COLONY job settings including typing error rate 0.001, mating system I with male and female polygamy, mating system II with inbreeding, run length medium and analysis method FL, and the rest of the settings were default. The main objective was full-sib and half-sib dyad detection and determination of the number of families within each analysed sample. Additionally, effective population size (Ne) was estimated using the sibship assignment method implemented in Colony (Wang 2009) and the average inbreeding coefficient at the 95% confidence interval.

## Results

### Age of the fish

Age analysis was performed to determine whether individual fish returning to the Vistula in 2017 and 2018 year could have come from artificial spawning in 2013. In 2017, among the 74 sea trout collected, the sea age was determined for 70 individuals (96%). Fish of sea age 1+ (58.5%) and 2+ (38.5%) dominated (Table S1). The age of smoltification was determined for 44 sea trout. Specimens of river age 2+ predominated. In the remaining cases, it was impossible to reliably determine the age of smoltification due to the regeneration of the scale centre, which is characteristic of farmed fish (Baglinière et al. 2020). The analysis of the age of the fish did not exclude, apart from one case (TAr8 individual), the possibility of the origin of these fish from spawning in 2013. In 2018, out of 381 collected sea trout, the sea age was determined for 296 fish (78%). The remaining cases are mostly fish from which no scales were collected. The largest group was sea trout in sea age 1 + (53.5%). The next group consisted of sea age 2 + (40%). Older individuals accounted for 4.7% while those at sea age 0 + only less than 2% (Table S2). The age of smoltification was considered reliable for 25% of the fish. River age 2 + dominated (Table S2).

### Parentage assignment for the release-recapture population

**Fig. 3:**
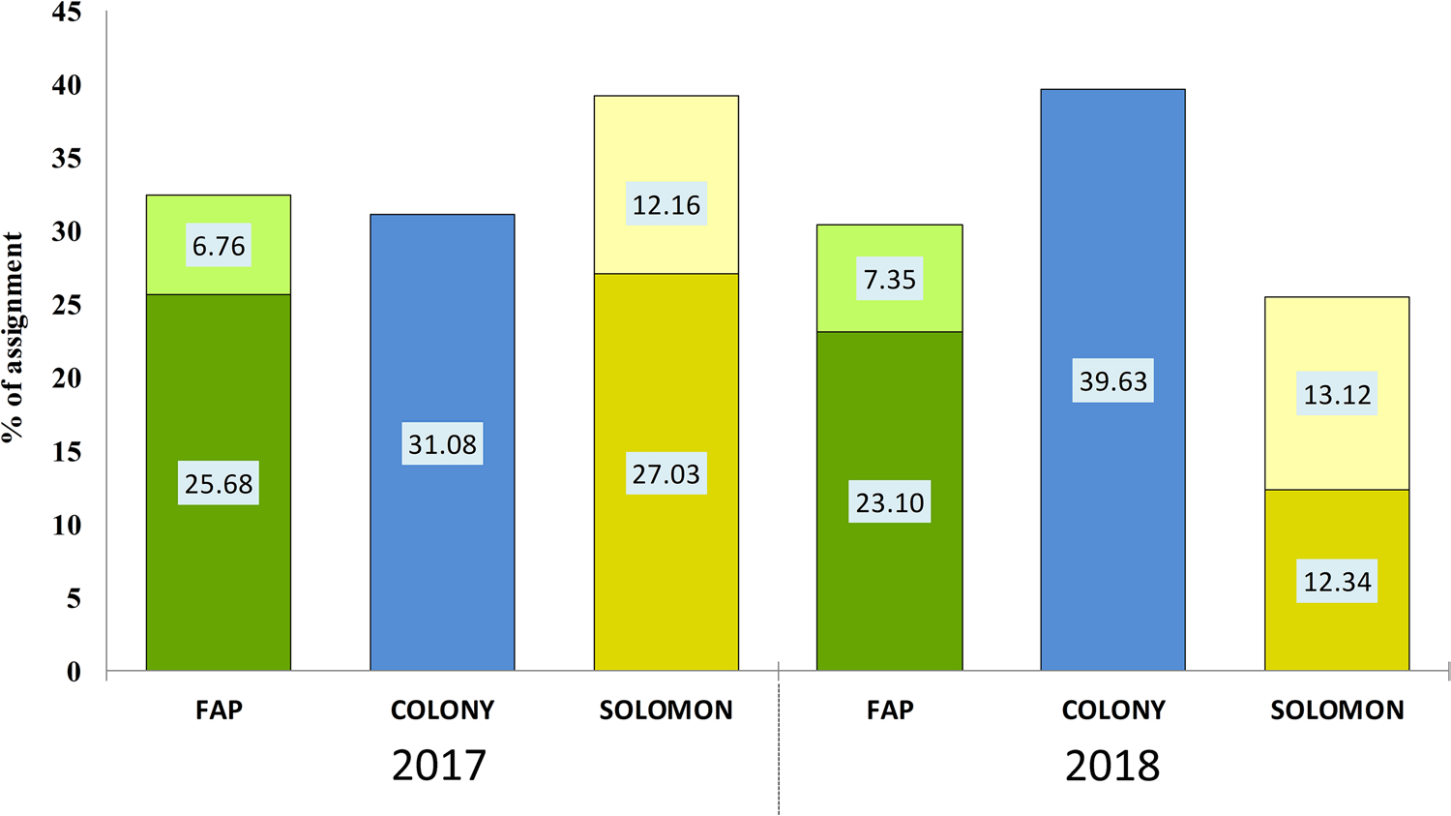
Assignment of sea trout from river fishing in 2017-2018 to parent stocks from 2013 determined by three algorithms. Computation using the FAP algorithm yielded results for error-free allocation (dark green) and one mismatched locus (light green). In the case of the SOLOMON algorithm, two probability thresholds are presented: *p* < 0.05 (light yellow) and *p* < 0.01 (dark yellow)

The analysis carried out using FAP indicated that the level of assigning offspring to parent stock from spawning in 2013 for fish collected in the Vistula in 2017 was 32.44%. Of these, 25.68% corresponded to the correct assignment of the complex genotype of the offspring and the indicated parental pair, while 6.76% corresponded one locus in the complex genotype showing no match. For the much more numerous samples from 2018, the level of the total allocation was 30.45%, of which 23.10% corresponded to error-free allocation and 7.35% to one mismatched locus (Fig. 3).

Using the COLONY algorithm, parents or parent pairs were assigned to 23 and 151 individuals caught in the Vistula in 2017 and 2018, respectively, constituting 31.08% and 39.63% of fish returning to the river. The analysis included only individuals for which the level of the likelihood of assignment was > 98% (Fig. 3). In the case of Bayesian analysis carried out using SOLOMON, two probability thresholds for rejecting the assignment were taken into account: For *p* < 0.01, the relationship for descendants returning to the river in 2017 and 2018 was confirmed for 27.03% and 12.34%, while for *p* < 0.05, these values were 12.16% and 13.12%, respectively (Fig. 3).

**Fig. 4:**
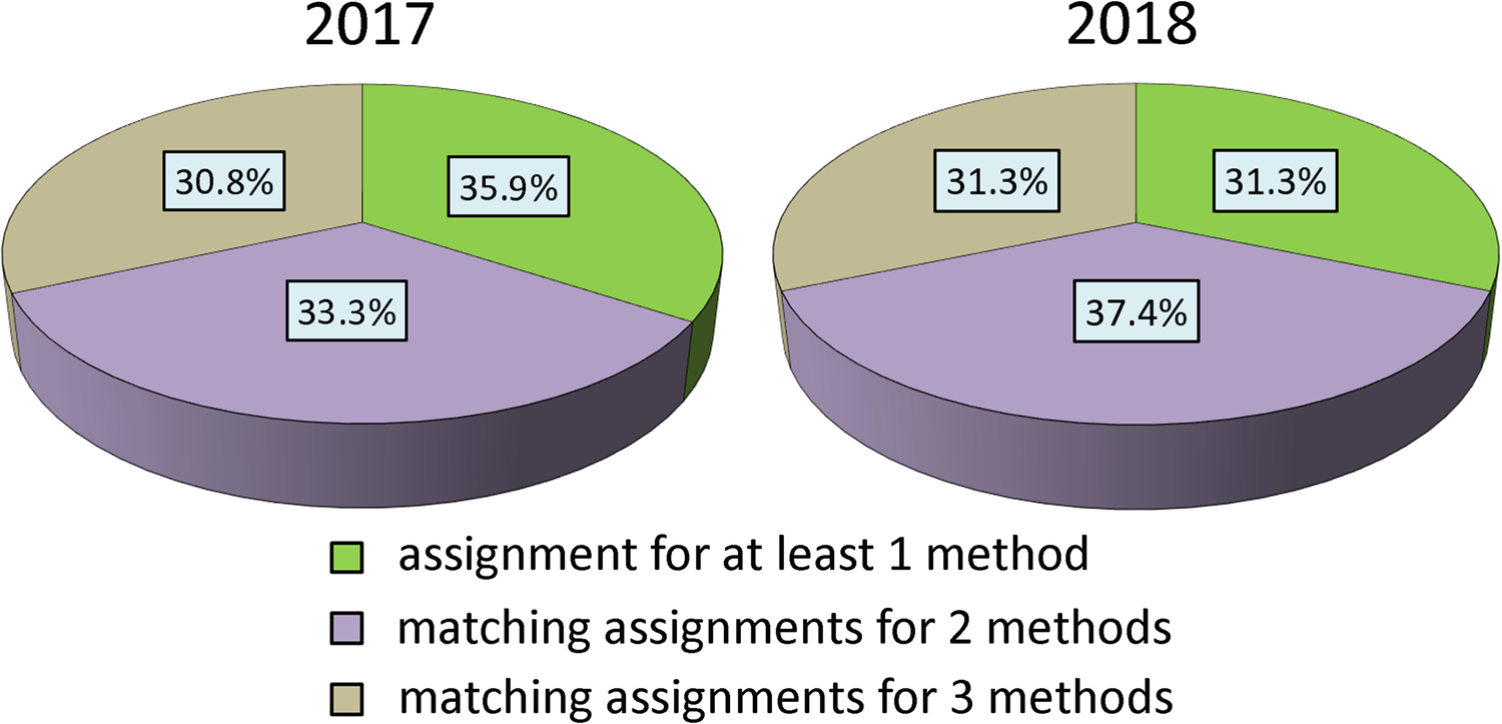
The percentage share of single and multiple assignments for returning sea trout from 2017 to 2018

Table S3 includes a list of all 221 fish assigned a parent pair or a specific parent based on the applied FAP, COLONY and SOLOMON algorithms. For 69 fish (2017 = 12, 2018 = 57), parent-offspring relationships were confirmed by all three algorithms (Fig. 4). Another 81 fish (2017 = 13, and 2018 = 68) were assigned parents using at least two algorithms. Assignments based on a single program were recorded for 71 fish returning to the Vistula in 2017-2018 (2017 = 14, 2018 = 57 fish; Fig. 4).

**Fig. 5:**
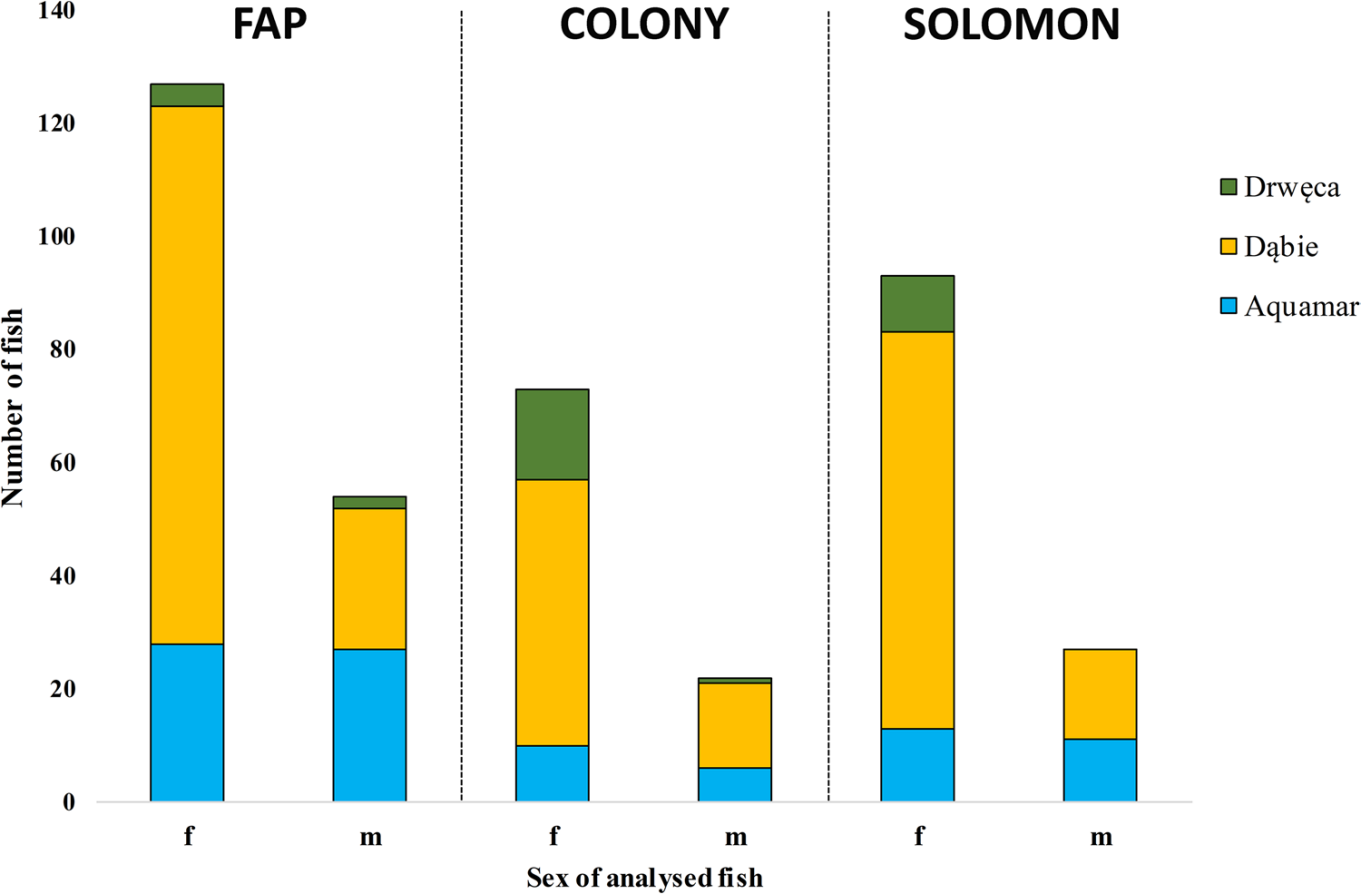
Share of males and females indicated as parents for fish returning to the Vistula in 2017-2018 identified by FAP, COLONY and SOLOMON algorithms

During analysis of parentage, the source of parental material (broodstocks/wild material collected in Drwęca) was consistently indicated by all three programs (except for three cases recorded for descendants of TC74, TCe94 and TCe147; Table S3). The largest number of identified descendants came from spawners from Dąbie fish farm (129 fish). Forty-six fish were assigned to spawners caught from Drwęca River, and 43 descendants came from spawners from Aquamar broodstock. The repeatability of assignment of parents to descendants indicated by at least two algorithms was also high, reaching 90%. There were cases, especially using the FAP algorithm, that yielded descendants associated with many potential parents. However, in these cases, the correct parent pair or individual parent was confirmed by computation using a different algorithm. The share of females and males recognised as sea trout that passed their genotype to the next generation upon spawning in 2013 differed depending on the algorithm used for calculation. The greatest number of parental females (F127) and males (M54) effectively involved in spawning was indicated by FAP. Parent numbers indicated by the other two algorithms did not differ substantially from each other (Fig. 5). In the vast majority of cases, a given individual was assigned to a single descendant, although in five cases a given parent was associated with many descendants (8-12).

### Familial structure and effective population size

Proportions of unrelated individuals within samples were very high (~ 99%), whereas full-sib proportions varied from 0.001 to 0.04%, and half-sib proportions ranged from 0.7 to 1.2 (Table 2 and Figure S1). The effective population size estimates ranged from 150 to 274 (Table 2). No major differences were observed between the tested parent stocks. The estimated inbreeding per generation was low and ranged between 0 and 3%.

**Table 2:** Results from the sibship assignment method employed by COLONY

Stock	Full-sib families	Unrelated dyads	Full-sib dyads	Half-sib dyads	Ne (CI95 L-U)	F_IS_
Aquamar	996	1 234 356	169 (0.001%)	8700 (0.7%)	274 (230–326)	0.017
Dąbie	382	185 521	60 (0.03%)	1908 (1.01%)	184 (150–229)	0.003
Lubicz	201	46 055	19 (0.04%)	582 (1.2%)	150 (117–189)	0.000

Full-sib and half-sib dyads are displayed both as absolute values and relative frequencies (brackets). Full-sib families concern all detections, including single detections. The last two columns are effective population size and inbreeding coefficient

## Discussion

The current research showed that the use of genetic methods based on analysis of relationships can be an effective alternative for estimating the effectiveness of mass stocking. However, for this to be the case, certain conditions must be met, such as genotyping all parental individuals and a sufficient number offspring, as well as using enough genetic markers.

A discussion follows on the reliability of the methods used to estimate kinship, and the impact of the obtained results on the management of the population of sea trout in the Vistula River.

### Effectiveness and reliability of kinship analysis

Overall parentage analysis based on genetic polymorphism uses exclusion-based and likelihood-based methods (Jones et al. 2010). When choosing a model for effective parentage analysis, certain conditions must be met. Most important is to have all parental genotypes and analyse large numbers of offspring. Herein, the data included all genotypes of all parents used in artificial spawning, and the number of offspring was large (> 100). Analyses must also enough polymorphic loci; this requirement was also met in the present work (Wąs-Barcz et al. 2017). and three complementary methods were applied and compared, making calculations more reliable.

Exclusion-based methods are simple and based on strait Mendelian inheritance. However, they are very sensitive to genotyping errors. The problem of genotyping errors can be solved by permitting a small number of mismatched alleles between offspring and parents (Vandeputte et al. 2006). During this study, estimations carried out with the FAP algorithm were based on the assumption of 0 and 1 for errors in determining the genotype, as suggested by the makers of the software. This allowed for assigning to an offspring, according to Mendelian genetics, a parent pair with 100% matching genotypes, or it was possible to differ one locus within the complex parental genotype. Applied methods based on likelihood (COLONY, SOLOMON) use quantitative Mendelian inheritance to calculate probability for various candidate relationships within a set, and relationships with the greatest inferred probability are selected. In the case of SOLOMON, the number of mismatched loci can be selected in the analysis (0 mismatches in this study), and this can solve problems related to genotyping errors. In turn, in the COLONY algorithm, individual genotyping errors (based on empirical data) are determined before analysis for all used loci.

The next issue is to apply the appropriate cut-off level. Choosing a threshold for accepting putative parent-offspring relationships should depend on the goals of the study and on weighing the relative risks between type I and type II errors (Christie et al. 2013). In the present study, the recommended level of 0.05 was used in the SOLOMON analysis, although the more restrictive level of 0.01 was also tested.

The methods used to detect parental pairs proved to be very effective, and the results obtained using individual algorithms were highly convergent. Depending on the method used, the share of sea trout from spawning in 2013 ranged from 26% to 40% in Vistula catches in 2017-2018. Additionally, among 150 assignments indicated by at least two programs, in the group of fish returning to the Vistula in 2017-2018, the agreement as to the source of origin of the assigned parent pair or a single parent was 98%.

### Management implications

The calculated share of fish from breeding stocks in the Vistula fishery indicates that a certain level of natural reproduction still occurs in the Vistula basin. It is interesting that this is consistent with the results of the analysis of catches from the southern Gdańsk Bay, where except ~ 30% of Pomeranian sea trout, a value of ~ 30% each of the Vistula and farmed Aquamar stocks was calculated using SNP microarray data (Bernaś et al. 2020). Such a high proportion of fish not identified as originated from stocking material from spawning performed in 2013 is somewhat surprising given the high stocking rate (ICES 2021). However, these 70% of fish in 2017 and 2018 could also be partly stocked in other years, and they are not necessarily wild fish. It should be noted that our results concern the share of farmed fish in river fisheries, and in order to compare it directly with Bernaś et al. (2020) results and fully explore the effectiveness of stocking, it would be necessary to include fish from sea fishing, although Bernaś et al. (2020) have shown that the proportion of farmed fish and potentially wild trout from Vistula was 1:1. This means that, in the river, we could expect about 50% of farmed fish that is not congruent with our data. Moreover, in recent years in the Vistula, the number of ascending sea trout has started to decrease drastically. Even 5 years ago, 1000–2000 sea trout passed through the fish counter located in the fish pass in Włocławek (ICES 2021). In the last few years, the number was much smaller, not exceeding 500 adult sea trout per year. Additionally, river fishing activity in the Vistula decreased significantly. The estuary section has been closed to fishing from 5 years, and the number of river fishermen has also decreased. However, this is not reflected in an increased population size. All these could be indirect evidences of the current reduction in stocking efficiency. Potential reasons for this are related to post-smolt mortality and the growing grey seal population at the mouth of the Vistula River (Mäntyniemi et al. 2012). The effect of stocking mostly with fish from closed breeding stocks is also a potential factor which magnifies their influence (e.g. Ruzzante et al. 2001; Hansen 2002).

In terms of assigning descendants to the used spawning stock, despite some discrepancies, related to differences in the share of each stock depending on the method (algorithm) used, the consistency of allocations to parents pair or single parent was very high. All three methods unanimously indicated that the largest share was confirmed for stock from Dąbie Hatchery (58.37% for considering the assignments of parental individuals by all three methods for 2017–2018). The share of the other two stocks was much lower reaching about 20% allocations in 2017–2018. The low proportion of descendants of the Drwęca spawning stock is understandable, given the much lower number of individuals of this stock released into the river during stocking. But it is difficult to explain the discrepancy between hatchery stocks, given that the average share of sea trout from these farms in the stocking of the Vistula River was similar. Possible explanation could be the existence of genetic differences between stocks, or epigenetic aspects resulting in slightly higher survival of sea trout from the Dąbie fish farm. The results of family structure analysis showed no visible differences between parent stocks, although slightly less relatedness was observed in Dąbie broodstock. Moreover, a significant improvement in variability and a reduction in the degree of kinship in the Aquamar broodstock was observed compared to the analysis in 2003 (Wąs and Bernaś 2016). This difference also does not result from the described phenomenon of weaker assignment of farmed fish than wild fish (Ford and Williamson 2010; Milot et al. 2013). Such a situation may occur with a clearly lower effective population size, which was not the case in our study; indeed, it was the opposite. At this point, it is worth mentioning that in general the Ne values were relatively low for all broodstocks. Taking into account the size of parental stocks and the historical size of the Vistula population, these values should generally be higher. This is probably the effect of the bottleneck phenomenon associated with a drastic decline in population size over the last 10 generations (Wąs and Bernaś 2016). The obtained results are slightly lower than those recommended for large breeding stocks (Tave 1999) and for wild populations (Frankham et al. 2014). They are also lower than those observed in the population of Pomeranian sea trout (Bernaś et al. 2014). Furthermore, the inbreeding values per generation are typical of those most frequently observed in breeding stocks, and even slightly lower (1–3%) (Ryman 1994; Tave 1999). Importantly, among the spawners from Lubicz, inbreeding was 0, which tells us that in this group the level of diversity is higher, and we had also individuals from natural spawning and various tributaries.

It is noticeable that in the case of Dąbie and Lubicz, the proportion of males to females effectively transmitting genes to the next generations is much lower, and this is also consistent with the natural process (skewed sex ratio) observed in migratory brown trout (e.g. Jonsson 1985; Bekkevold et al. 2004). However, in the case of Aquamar, it is close to a 1:1 ratio, and it was observed for all methods (including those calculating the probability for one parent). This is the result of intentional breeding and the use of large numbers of males to fertilise individual batches of eggs in the Aquamar hatchery and, hence, the higher values of the effective population size in this broodstock (Tave 1999).

## Conclusions

Several conclusions can be drawn based on the findings of this study. First, our work shows that the methods based on parentage analysis were effective for estimating mass stocking success. Therefore, we are currently conducting similar analyses on several southern Baltic rivers to estimate the effectiveness of individual stocking in addition to marking with RFID tags (ICES 2021). The share of sea trout from spawning in 2013 in Vistula sea trout catches from 2017 to 2018 reached ~ 30%. The results also indicated that a certain level of natural reproduction still occurs in the Vistula River.

## Supplementary information

Supplementary file 1

Supplementary file 2
