# Correction to: Parentage‑based tagging and parentage analyses of stocked sea trout in Vistula River commercial catches

**DOI:** 10.1007/s13353-023-00751-1

**Published:** 2023-02-10

**Authors:** Anna Wąs‑Barcz, Rafał Bernaś

**Affiliations:** 1 Department of Fisheries Resources, National Marine Fisheries Research Institute, Kołłątaja 1, 81‑332 Gdynia, Poland; 2 Department of Migratory Fish, National Inland Fisheries Research Institute, Rutki 49, 83‑330 Żukowo, Poland

**Correction to: Journal of Applied Genetics (2023)**

**https://doi.org/10.1007/s13353-023-00749-9**

Originally, the article was published with error. In the Abstract, the second line should be "in the twentieth century" instead of "in the twenty-first century".

The original article has been corrected.

